# The International Stroke Trial database

**DOI:** 10.1186/1745-6215-12-101

**Published:** 2011-04-21

**Authors:** Peter AG Sandercock, Maciej Niewada, Anna Członkowska

**Affiliations:** 1 Department of Clinical Neurosciences, University of Edinburgh, Department of Clinical Neurosciences, Western General Hospital, Edinburgh EH4 2XU, UK; 2 Department of Clinical and Experimental Pharmacology, Warsaw Medical University, Poland, Krakowskie Przedmieście 26/28, 00-927 Warsaw, Poland; 3 2nd Department of Neurology, Institute of Psychiatry and Neurology, 9 Sobieskiego, 02-957 Warsaw, Poland

## Abstract

**Background:**

We aimed to make individual patient data from the International Stroke Trial (IST), one of the largest randomised trials ever conducted in acute stroke, available for public use, to facilitate the planning of future trials and to permit additional secondary analyses.

**Methods:**

For each randomised patient, we have extracted data on the variables assessed at randomisation, at the early outcome point (14-days after randomisation or prior discharge) and at 6-months and provide them as an analysable database.

**Results:**

The IST dataset includes data on 19 435 patients with acute stroke, with 99% complete follow-up. Over 26.4% patients were aged over 80 years at study entry. Background stroke care was limited and none of the patients received thrombolytic therapy.

**Conclusions:**

The IST dataset provides a source of primary data which could be used for planning further trials, for sample size calculations and for novel secondary analyses. Given the age distribution and nature of the background treatment given, the data may be of value in planning trials in older patients and in resource-poor settings.

## Background

The International Stroke Trial (IST) was conducted between 1991 and 1996 (including the pilot phase between 1991 and 1993). It was a large, prospective, randomised controlled trial, with 100% complete baseline data and over 99% complete follow-up data. The aim of the trial was to establish whether early administration of aspirin, heparin, both or neither influenced the clinical course of acute ischaemic stroke [1].

## Methods

The study had a prospective, randomised, open treatment, blinded outcome (PROBE) design. The inclusion criteria were: clinical diagnosis of acute ischaemic stroke, with onset within the previous 48 hours and no clear indication for, or clear contraindication to, treatment with aspirin or subcutaneous heparin. Unlike many stroke trials of that era (and subsequently), the study did not set an upper age limit. Patients were to have a CT brain scan to confirm the diagnosis of stroke, and this was to be done before randomisation if at all possible. To enter a patient in the study, the clinician telephoned a central randomisation service (at the Clinical Trial Service Unit, Oxford) during this telephone call, the baseline variables were entered and checked, and once validated, the computer allocated the treatment and the telephonist then informed the clinician. The patients and treating clinicians were not blinded to the treatment given. Early outcome data were collected by the treating physician who completed a follow-up form at 14 days, death or hospital discharge (whichever occurred first). This form recorded data on events in hospital within 14 days, and the doctor's opinion on the final diagnosis of the initial event that led to randomisation. These unblinded data, may therefore be subject to some degree of bias. The primary outcome was the proportion of patients who were either dead or dependent on other people for activities of daily living at six months after randomisation. This outcome was collected by postal questionnaire mailed directly to the patient, or (in Italy) by telephone interview of the patient by a trained researcher, blinded to treatment allocation. The primary outcome was therefore assessed - as far as practicable - blind to treatment allocation and hence should be free from bias. We re-checked the data set for inaccuracies and inconsistencies and extracted data on the variables assessed at randomisation, and at the two outcome assessment points: at 14-days after randomisation, death or prior hospital discharge (whichever occurred first) and at 6-months.

## Results

Consent for publication of raw data was not obtained from participants. Consent for participation in the trial was obtained from all subjects or from an appropriate proxy, according to the procedures approved by relevant national and local hospital ethics committees (or Institutional Review Boards [IRB]). These patients were treated 15-20 years ago, and many have died. The dataset (see additional file 1 - IST_data.csv) is fully anonymous in a manner that can easily be verified by any user of the dataset. Patients and hospitals are identified only by an anonymous code; there are no identifying data such as name, address or social security numbers; patient age has been rounded to the nearest whole number. In our view, publication of the dataset clearly presents no material risk to confidentiality of study participants.

The dataset includes the following baseline data: age, gender, time from onset to randomisation, presence or absence of atrial fibrillation (AF), aspirin administration within 3 days prior to randomisation, systolic blood pressure at randomisation, level of consciousness and neurological deficit. The deficits were classified as one of the Oxfordshire Community Stroke Project (OCSP) categories: total anterior circulation syndrome (TACS), partial anterior circulation syndrome (PACS), posterior circulation syndrome (POCS) and lacunar syndrome (LACS). We extracted events within 14 days on: the occurrence of recurrent stroke, pulmonary embolism, and death (date and cause of death). At 6 months we extracted: degree of recovery, place of residence and current use of antiplatelet or anticoagulant drugs and death (date and cause of death). The cause of death was classified as: due to initial stroke, recurrent ischaemic stroke, recurrent haemorrhagic stroke, pneumonia, coronary artery disease, pulmonary embolism, other vascular cause or a nonvascular cause. Patients were assigned to one of 6 categories according to the place of residence at 6 months following stroke: own home, relatives home, residential care, nursing home, other hospital departments or unknown. The variables extracted are listed with a brief description of each in Tables 1, 2 and 3. Nineteen thousand four hundred and thirty five patients from 467 hospitals in 36 countries were randomised within 48 hours of symptoms onset, of whom 13020 had a CT before randomisation, 5569 were first scanned after randomisation and 846 were not scanned at all. Five thousand one hundred thirty two (26.4%) were aged over 80 years at study entry. Given that 5569 patients were first scanned after randomisation, and 846 were not scanned at all, the 'final diagnosis' is somewhat imprecise. However, since the analysis was by intention to treat, all participants were retained in the analysis, irrespective of the final diagnosis. The numbers of patients with each final diagnosis are given in Table 4. Whilst the 'final diagnosis variable' is of some interest, it may be influenced by events occurring after randomisation, so for any future analyses, the least biased assessment of the patient characteristics is that recorded at baseline, before randomisation.

**Table 1:** Country codes used in International Stroke Trial.

Country	Code
Albania	43

Argentina	29

Australia	01

Belgium	03

Brazil	42

Bulgaria	04

Canada	05

Chile	06

Czech Republic	07

Denmark	08

Ireland	09

Finland	10

France	11

Georgia	32

Germany	12

Greece	31

Hong Kong	30

Hungary	36

India	37

Indonesia	41

Israel	13

Italy	14

Japan	38

Latvia	39

Malaysia	40

Netherlands	15

New Zealand	16

Norway	17

Poland	18

Portugal	19

Romania	33

Singapore	34

Slovak Republic	44

Slovenia	20

South Africa	21

Spain	22

Sri Lanka	23

Sweden	24

Switzerland	25

Thailand	26

Turkey	35

UK	27

USA	28

**Table 2:** Variables names and comments.

Randomisation data	
HOSPNUM	Hospital number

RDELAY	Delay between stroke and randomisation in hours

RCONSC	Conscious state at randomisation (F - fully alert, D - drowsy, U - unconscious)

SEX	M = male; F = female

AGE	Age in years

RSLEEP	Symptoms noted on waking (Y/N)

RATRIAL	Atrial fibrillation (Y/N); not coded for pilot phase - 984 patients

RCT	CT before randomisation (Y/N)

RVISINF	Infarct visible on CT (Y/N)

RHEP24	Heparin within 24 hours prior to randomisation (Y/N)

RASP3	Aspirin within 3 days prior to randomisation (Y/N)

RSBP	Systolic blood pressure at randomisation (mmHg)

RDEF1	Face deficit (Y/N/C=can't assess)

RDEF2	Arm/hand deficit (Y/N/C=can't assess)

RDEF3	Leg/foot deficit (Y/N/C=can't assess)

RDEF4	Dysphasia (Y/N/C=can't assess)

RDEF5	Hemianopia (Y/N/C=can't assess)

RDEF6	Visuospatial disorder (Y/N/C=can't assess)

RDEF7	Brainstem/cerebellar signs (Y/N/C=can't assess)

RDEF8	Other deficit (Y/N/C=can't assess)

STYPE	Stroke subtype (TACS/PACS/POCS/LACS/OTH=other)

RDATE	Year and month of randomisation (yyyy-mm)

HOURLOCAL	Local time - hours (99-missing data) of randomisation

MINLOCAL	Local time - minutes (99-missing data) of randomisation

DAYLOCAL	Estimate of local day of week; 1 - Sunday, 2-Monday, 3-Tuesday, 4-Wednesday, 5-Thursday, 6-Friday, 7-Saturday

RXASP	Trial aspirin allocated (Y/N)

RXHEP	Trial heparin allocated (M/L/N). The terminology for the allocated dose of unfractioned heparin changed slightly from the pilot to the main study. Patients were allocated either 12500 units subcutaneously twice daily (coded as H in the pilot and M in the main trial), 5000 units twice daily (coded as L throughout) or to 'avoid heparin' (coded as N throughout).

**Data collected on 14 day/discharge form about treatments given in hospital**	

DASP14	Aspirin given for 14 days or till death or discharge (Y/N/U=unknown)

DASPLT	Discharged on long term aspirin (Y/N/U=unknown)

DLH14	Low dose heparin given for 14 days or till death/discharge (Y/N/U=unknown)

DMH14	Medium dose heparin given for 14 days or till death/discharge (Y/N/U=unknown)

DHH14	Medium dose heparin given for 14 days etc in pilot (combine with above; Y/N)

ONDRUG	Estimate of time in days on trial treatment

DSCH	Non trial subcutaneous heparin (Y/N/U=unknown)

DIVH	Non trial intravenous heparin (Y/N/U=unknown)

DAP	Non trial antiplatelet drug (Y/N/U=unknown)

DOAC	Other anticoagulants (Y/N/U=unknown)

DGORM	Glycerol or manitol (Y/N/U=unknown)

DSTER	Steroids (Y/N/U=unknown)

DCAA	Calcium antagonists (Y/N/U=unknown)

DHAEMD	Haemodilution (Y/N/U=unknown)

DCAREND	Carotid surgery (Y/N/U=unknown)

DTHROMB	Thrombolysis (Y/N/U=unknown)

DMAJNCH	Major non-cerebral haemorrhage (Y/N/U=unknown)

DMAJNCHD	Date of above (days elapsed from randomisation)

DMAJNCHX	Comment on above

DSIDE	Other side effect (Y/N/U=unknown)

DSIDED	Date of above (days elapsed from randomisation)

DSIDEX	Comment on above

**Final diagnosis of initial event**	

DDIAGISC	Ischaemic stroke (Y/N/U=unknown)

DDIAGHA	Haemorrhagic stroke (Y/N/U=unknown)

DDIAGUN	Indeterminate stroke (Y/N/U=unknown)

DNOSTRK	Not a stroke (Y/N/U=unknown)

DNOSTRKX	Comment on above

** Recurrent stroke within 14 days**	

DRSISC	Ischaemic recurrent stroke (Y/N/U=unknown)

DRSISCD	Date of above (days elapsed from randomisation)

DRSH	Haemorrhagic stroke (Y/N/U=unknown)

DRSHD	Date of above (days elapsed from randomisation)

DRSUNK	Unknown type (Y/N/U=unknown)

DRSUNKD	Date of above (days elapsed from randomisation)

**Other events within 14 days**	

DPE	Pulmonary embolism; (Y/N/U=unknown)

DPED	Date of above (days elapsed from randomisation)

DALIVE	Discharged alive from hospital (Y/N/U=unknown)

DALIVED	Date of above (days elapsed from randomisation)

DPLACE	Discharge destination (A-Home/B-Relatives home/C-Residential care/D-Nursing home/E-Other hospital departments/U-Unknown)

DDEAD	Dead on discharge form (Y/N/U=unknown)

DDEADD	Date of above (days elapsed from randomisation); NOTE: this death is not necessarily within 14 days of randomisation

DDEADC	Cause of death (1-Initial stroke/2-Recurrent stroke (ischaemic or unknown)/3-Recurrent stroke (haemorrhagic)/4-Pneumonia/5-Coronary heart disease/6-Pulmonary embolism/7-Other vascular or unknown/8-Non-vascular/0-unknown)

DDEADX	Comment on death

**Data collected at 6 months**	

FDEAD	Dead at six month follow-up (Y/N/U=unknown)

FLASTD	Date of last contact (days elapsed from randomisation)

FDEADD	Date of death (days elapsed from randomisation); NOTE: this death is not necessarily within 6 months of randomisation

FDEADC	Cause of death (1-Initial stroke/2-Recurrent stroke (ischaemic or unknown)/3-Recurrent stroke (haemorrhagic)/4-Pneumonia/5-Coronary heart disease/6-Pulmonary embolism/7-Other vascular or unknown/8-Non-vascular/0-unknown)

FDEADX	Comment on death

FRECOVER	Fully recovered at 6 month follow-up (Y/N/U=unknown)

FDENNIS	Dependent at 6 month follow-up (Y/N/U=unknown)

FPLACE	Place of residance at 6 month follow-up (A-Home/B-Relatives home/C-Residential care/D-Nursing home/E-Other hospital departments/U-Unknown)

FAP	On antiplatelet drugs at six month follow-up (Y/N/U=unknown)

FOAC	On oral anticoagulants at six month follow-up (Y/N/U=unknown)

**Other data and derived variables**	

FU1_RECD	Date discharge form received (days elapsed from randomisation)

FU2_DONE	Date 6 month follow-up done (days elapsed from randomisation)

COUNTRY	Abbreviated country code

CNTRYNUM	Country code (see Table 1)

FU1_COMP	Date discharge form completed (days elapsed from randomisation)

NCCODE	Coding of compliance (see Table 3)

CMPLASP	Compliant for aspirin (N/Y)

CMPLHEP	Compliant for heparin (N/Y)

ID	Indicator variable for death (1 = died; 0 = did not die)

TD	Time of death or censoring in days

EXPDD	Predicted probability of death/dependence at 6 month

EXPD6	Predicted probability of death at 6 month

EXPD14	Predicted probability of death at 14 days

SET14D	Know to be dead or alive at 14 days (1 = Yes, 0 = No); this does not necessarily mean that we know outcome at 6 months - see OCCODE for this

ID14	Indicator of death at 14 days (1 = Yes, 0 = No)

OCCODE	Six month outcome (1-dead/2-dependent/3-not recovered/4-recovered/0 or 9 - missing status

** Indicator variables for specific causes of death**	

DEAD1	Initial stroke (1 = Yes, 0 = No)

DEAD2	Reccurent ischaemic/unknown stroke (1 = Yes, 0 = No)

DEAD3	Reccurent haemorrhagic stroke (1 = Yes, 0 = No)

DEAD4	Pneumonia (1 = Yes, 0 = No)

DEAD5	Coronary heart disease (1 = Yes, 0 = No)

DEAD6	Pulmonary embolism (1 = Yes, 0 = No)

DEAD7	Other vascular or unknown (1 = Yes, 0 = No)

DEAD8	Non vascular (1 = Yes, 0 = No)

H14	Cerebral bleed/heamorrhagic stroke within 14 days; this is slightly wider definition than DRSH and is used for analysis of cerebral bleeds; (1 = Yes, 0 = No)

ISC14	Indicator of ischaemic stroke within 14 days (1 = Yes, 0 = No)

NK14	Indicator of indeterminate stroke within 14 days (1 = Yes, 0 = No)

STRK14	Indicator of any stroke within 14 days (1 = Yes, 0 = No)

HTI14	Indicator of haemorrhagic transformation within 14 days (1 = Yes, 0 = No)

PE14	Indicator of pulmonary embolism within 14 days (1 = Yes, 0 = No)

DVT14	Indicator of deep vein thrombosis on discharge form (1 = Yes, 0 = No)

TRAN14	Indicator of major non-cerebral bleed within 14 days (1 = Yes, 0 = No)

NCB14	Indicator of any non-cerebral bleed within 14 days (1 = Yes, 0 = No)

**Table 3:** Provisional categories for non compliance (NCCODE).

1.	Should not have been randomised
2.	Refused treatment

3.	Initial event not a stroke

4.	Haemorrhagic stroke

5.	Non compliers

6.	Discharged after 14 days

7.	Discharged up to 14 days

8.	Died prior to receiving the study drug(s)

9.	Died after receiving the study drug(s)

10.	Recurrent stroke/pulmonary embolism

11.	Clinical decision

11a.	Suspected abnormality

11b.	Withdrawn as dying

11c.	Pre-existing condition

11d.	Stated abnormal PTT

11e.	Stated surgery

11f.	Stated atrial fibrillation

12.	Administration problem

13.	Missed out more than 3 doses

14.	Side effect

14a.	Refused treatment

14b.	Discharged

14c.	Administration problem

14d.	Clinical decision

14e.	Recurrent stroke

14f.	Haemorrhagic stroke

**Table 4:** Final diagnosis of initial event.

	Number
Ischaemic stroke	17398

Haemorrhagic stroke	599

Definite stroke, pathological type unknown	992

Not a stroke	420

Uncertain diagnosis	26

Total	19435

To restrict analyses to cases of definite ischaemic stroke, confirmed at the time of trial entry, the variable denoting whether CT had been performed before entry (RCT) should = Y and the final diagnosis should also be ischaemic (DDIAGISC=Y).

Please note that, in the original 1997 Lancet report on the trial [1], figures two a and two b reported the effects of allocation to aspirin and to heparin on the primary outcome, subdivided by various baseline characteristics and by the final diagnosis. The numbers of patients with each pathological type of stroke are somewhat different to the numbers above, because they relate to the number of patients with complete 6 month follow-up data, whereas the numbers above relate to all randomised patients.

### Anonymisation

As recommended by *Hrynaszkiewicz et al. *[2] we have removed all direct and indirect identifiers from the database. We therefore present patient's age rounded to the nearest whole number of years. Time of admission to hospital (a potential identifier) was not recorded. Dates of events occurring post randomisation have been converted to the number of days from randomisation. The time variables that were recorded (see below) referred to time of randomisation in the trial (i.e. the time at which the system generated the treatment allocation), not time of admission to hospital, a variable, that - in our view - would not help identify the patient.

## Discussion

This large data set, with very complete follow-up, includes a very broad range of acute stroke patients with a uniquely large number of very elderly patients, and so may be useful to researchers planning future research studies. Users of the dataset should be aware that the study was conducted at a time when stroke unit care was not widely available and thrombolytic therapy was used rarely (and none of the included patients received it) [3]. Thus, the background stroke care for the included subjects, while not typical of present-day acute stroke care [4], is perhaps more typical of current stroke care in resource poor settings [5]. Given that the developing world faces a future epidemic of non-communicable diseases, including stroke [5], these data may therefore prove particularly valuable for planning future trials in resource-poor settings. In the developed world, the proportion of the general population who are 'very elderly' is rapidly increasing. Older people have been substantially under-represented in stroke trials to date [6], so we hope the large number of patients aged over 80 in this data set could also facilitate planning of trials in the 'older old'.

The publication of raw datasets such as the IST's may offer wholly unanticipated benefits to the wider research community. For example, the dataset was licensed to an independent statistical group who used the data to estimate the size and direction of biases introduced when non-randomised comparisons were made and the differences between direct and indirect comparisons. This empirical work led to two important publications on the topic [7,8]. Such additional benefits, realised long after the original trial was completed, are a further clear indication of the value of opening access to such datasets.

## Competing interests

The trial was designed, conducted, analyzed, and reported independently of all sponsors. P.S., M.N., A.C. have received honoraria and travel expenses to lecture at conferences and pharmaceutical advisory meetings, but neither holds any consultancy with, or financial interest in, a pharmaceutical company, nor are they aware of any other potential conflict of interest.

## Note for users of the data set

The authors ask that any publications arising from the use of this dataset acknowledges the source of the dataset, its funding and the collaborative group that collected the data.

## Authors' contributions

PS was the Chief Investigator of the IST responsible for the overall design, conduct and presentation of the study results. MN and AC were involved in preparation of database and data dictionary for publication and help to draft the manuscript. All authors read and approved the final manuscript.

## Sources of Funding

The study was principally funded by the UK Medical Research Council, the UK Stroke Association, and the European Union BIOMED-1 program. Limited support for collaborators' meetings and travel was provided by Eli Lilly, Sterling Winthrop (now Bayer USA), Sanofi, and Bayer UK. Follow-up in Australia was supported by a grant from the National Heart Foundation and in Canada by a Nova Scotia Heart and Stroke Foundation grant. Czech Republic IST was supported by a grant from the IGA Ministry of Health. India IST was supported by the McMaster INCLEN program and the All India Institute of Medical Sciences. The IST in New Zealand was funded by the Julius Brendel Trust and the Lottery Grants Board. In Norway, the IST was supported by the Norwegian Council on Cardiovascular Disease and Nycomed (for insurance).

## Supplementary Material

Additional file 1 **Database with information completed in IST**. Click here for file
