# The International Stroke Trial database

**DOI:** 10.1186/1745-6215-13-24

**Published:** 2012-03-06

**Authors:** Peter AG Sandercock, Maciej Niewada, Anna Członkowska

**Affiliations:** 1 Department of Clinical Neurosciences, University of Edinburgh, Department of Clinical Neurosciences, Western General Hospital, Edinburgh EH4 2XU, UK; 2 Department of Clinical and Experimental Pharmacology, Warsaw Medical University, Poland, Krakowskie Przedmieście 26/28, 00-927 Warsaw, Poland; 3 2nd Department of Neurology, Institute of Psychiatry and Neurology, 9 Sobieskiego 02-957, Warsaw, Poland

## Correction

Following the publication of our article [1] we became aware of errors introduced in the following variables which are all dates of events variables, i.e.:

DMAJNCHD: Date of above (days elapsed from randomisation)

DSIDED: Date of above (days elapsed from randomisation)

DRSISCD: Date of above (days elapsed from randomisation)

DRSHD: Date of above (days elapsed from randomisation)

DRSUNKD: Date of above (days elapsed from randomisation)

DPED: Date of above (days elapsed from randomisation)

DALIVED: Date of above (days elapsed from randomisation)

DDEADD: Date of above (days elapsed from randomisation)

FLASTD: Date of last contact (days elapsed from randomisation)

FDEADD: Date of death (days elapsed from randomisation)

FU1_RECD: Date discharge form received (days elapsed from randomisation)

FU2_DONE: Date 6 month follow-up done (days elapsed from randomisation)

FU1_COMP: Date discharge form completed (days elapsed from randomisation).

These errors, that were introduced in the process of anonymisation, have now been corrected in the final, double checked version of the dataset, which is also available at the University of Edinburgh repository (see http://datashare.is.ed.ac.uk/). Both this corrected and the erroneous version of the dataset are available to allow comparison with any previous analyses of the data. The corrected submission states clearly that it supersedes an earlier one.
